# The Induction of Combined Hyperthermal Ablation Effect of Irreversible Electroporation with Polydopamine Nanoparticle-Coated Electrodes

**DOI:** 10.3390/ijms25084317

**Published:** 2024-04-13

**Authors:** Sung-Min Jeon, Enkhzaya Davaa, Ratchapol Jenjob, Chiravoot Pechyen, Sitakan Natphopsuk, Seok Jeong, Hye Jin Yoo, Su-Geun Yang

**Affiliations:** 1 Department of Biomedical Science, BK21 FOUR Program in Biomedical Science and Engineering, Inha University College of Medicine, Incheon 22212, Republic of Korea; 2 Inha Institute of Aerospace Medicine, Inha University College of Medicine, Incheon 22332, Republic of Korea; 3 Thammasat University Center of Excellence in Modern Technology and Advanced Manufacturing for Medical innovation, Thammasat University, Pathumthani 12120, Thailand; 4 Department of Materials and Textile Technology, Faculty of Science and Technology, Thammasat University, Pathumthani 12120, Thailand; 5 Chulabhorn International College of Medicine, Thammasat University, Pathumthani 12120, Thailand; 6 Division of Gastroenterology, Inha University Hospital, Inha University College of Medicine, Incheon 22332, Republic of Korea

**Keywords:** irreversible electroporation (IRE), polydopamine nanoparticles, hyperthermal effect, cancer ablation

## Abstract

Irreversible electroporation (IRE) is a prominent non-thermal ablation method widely employed in clinical settings for the focal ablation therapy of solid tumors. Utilizing high-voltage, short-duration electric pulses, IRE induces perforation defects in the cell membrane, leading to apoptotic cell death. Despite the promise of irreversible electroporation (IRE) in clinical applications, it faces challenges concerning the coverage of target tissues for ablation, particularly when compared to other thermal ablation therapies such as radiofrequency ablation, microwave ablation, and cryoablation. This study aims to investigate the induced hyperthermal effect of IRE by applying a polydopamine nanoparticle (Dopa NP) coating on the electrode. We hypothesize that the induced hyperthermal effect enhances the therapeutic efficacy of IRE for cancer ablation. First, we observed the hyperthermal effect of IRE using Dopa NP-coated electrodes in hydrogel phantom models and then moved to in vivo models. In particular, in in vivo animal studies, the IRE treatment of rabbit hepatic lobes with Dopa NP-coated electrodes exhibited a two-fold higher increase in temperature (ΔT) compared to non-coated electrodes. Through a comprehensive analysis, we found that IRE treatment with Dopa NP-coated electrodes displayed the typical histological signatures of hyperthermal ablation, including the disruption of the hepatic cord and lobular structure, as well as the infiltration of erythrocytes. These findings unequivocally highlight the combined efficacy of IRE with Dopa NPs for electroporation and the hyperthermal ablation of target cancer tissues.

## 1. Introduction

Irreversible electroporation (IRE) emerges as an innovative and promising therapy for cancer ablation, introducing a distinctive approach to combating tumors. This technique employs micro- to millisecond electrical pulses directed at tumors, inducing cell necrosis through the permeabilization of cell membranes [1]. The key characteristic of IRE lies in its capacity to bring about irreversible disruption to the integrity of cell membranes and subsequent cell death, differentiating it from established thermal ablation methods like radiofrequency, microwave, and laser [2,3]. Notably, irreversible electroporation (IRE) stands out as a groundbreaking technique for achieving tissue death without recourse to thermal energy or heating [4,5]. This unique characteristic renders IRE particularly advantageous, as it obviates significant considerations related to the dissipation of thermal energy. Consequently, there are no collateral heat injuries to surrounding tissues and no risk of heat sink effects [6]. Nevertheless, it is essential to acknowledge certain limitations associated with IRE. One notable constraint is its inability to comprehensively cover the entire cancer region, primarily due to the current lack of a robust method for precisely controlling the direction of electricity. The irregular shape of tumor masses further complicates the application of IRE, and variations in tissue conductivity present additional challenges for achieving uniform and effective treatments [7]. It is widely acknowledged that the ablation zone for irreversible electroporation (IRE) is relatively small, making it challenging to cover large solid tumors effectively [8]. The induction of mild thermal ablation with IRE may increase the therapeutic availability of IRE for cancer treatment.

Recent studies have demonstrated the combined treatment of IRE with other therapeutic modalities, displaying a synergistic effect for cancer therapy. Our group reported that the combination treatment of IRE and gold-silica nanoparticles increased cell membrane permeability, cellular reactive oxygen species (ROS), and the lipid peroxidation of EMT-6 cells [9].

Polydopamine is a highly biocompatible bioinspired material that incorporates many functional groups such as catechol, amine, and imine that can be used to bind specific molecules or to load transition metal ions [10,11,12,13]. One of valuable features of polydopamine is that polydopamine can be easily deposited on various substrates with good control of the film thickness [14,15]. Singler and his research group reported that polydopamine functional coatings with AgNO_3_ greatly enhanced the electroconductivity of polyethylene terephthalate substrate [16]. Liu and his group reported that electropolymerized polydopamine enhanced the conductivity of polypyrrole hydrogels [17]. Based on these previous reports, we supposed that electroconductive polydopamine and polydopamine-derived nanoparticles may dissolve the clinical limitations of IRE.

**Figure 1:**
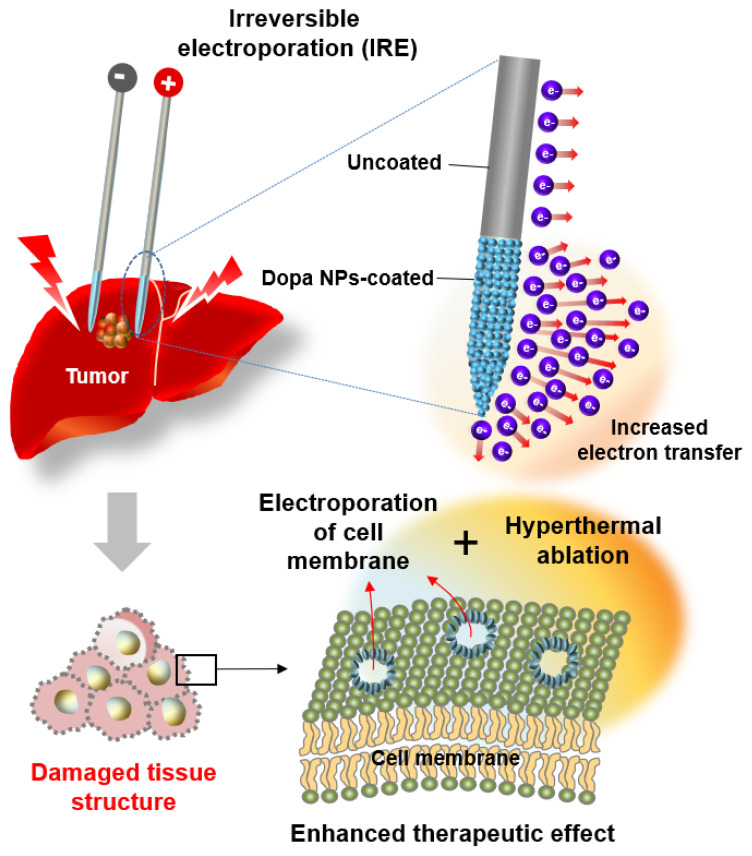
Schematic illustration of the enhanced thermal ablation effect of irreversible electroporation with dopa nanoparticle-coated electrodes.

This study focuses on the hypothesis that polydopamine nanoparticles (Dopa NPs) could improve electron transfer when applied to the surface coating of IRE electrodes, ultimately causing tumor damage to tumor cells through nanopore formation and hyperthermal effects (Figure 1). The clinical and preclinical efficacies of cancer hyperthermal therapy have been extensively demonstrated through various approaches. In particular, polydopamine nanoparticles have garnered significant attention for their application in the photo-induced thermal therapy of solid tumors [18,19]. However, the electrothermal therapeutic application of polydopamine nanoparticles has not been studied thus far. To validate this conjecture, we engineered IRE electrodes with a coating of Dopa NPs and conducted a comparative analysis with uncoated electrodes. In vitro assessments of the heat generation of Dopa NP-coated IRE electrodes using phantom models were performed. And then in vivo treatments were performed on the rabbit hepatic lobes in order to evaluate the hyperthermal-induced tissue damage, i.e., disruption of the hepatic cord and lobular structure and infiltration of erythrocytes, and comprehensively validate our hypothesis.

## 2. Results and Discussion

### 2.1. Synthesis and Characteristics of Dopa NPs

**Figure 2:**
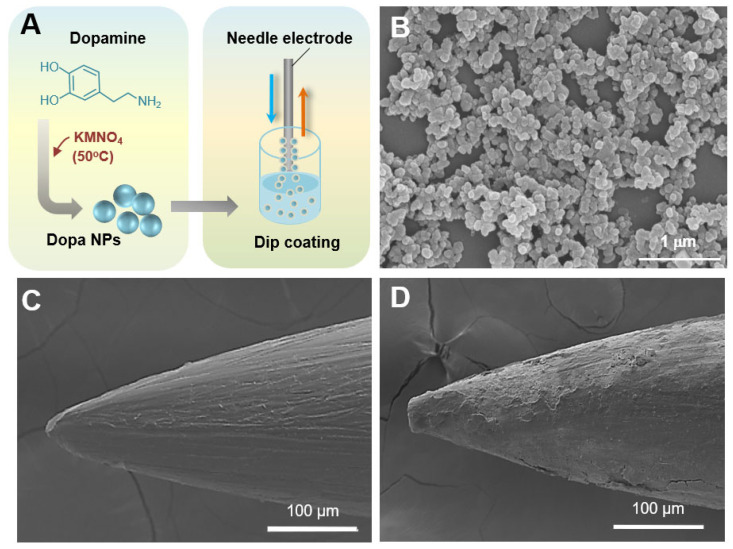
Dopa NP-coated IRE electrodes. (**A**) Schematic diagram of Dopa NP synthesis (**left**) and dip coating of electrodes into Eudragit^®^ S100-based polymeric solution (**right**). (**B**) SEM image of the synthesized Dopa NPs (×30,000). (**C**) SEM image of uncoated electrode (×350). (**D**) SEM image of Dopa NP-coated electrode (×350).

KMnO_4_, an oxidizing agent, was used for the preparation of Dopa NPs through the oxidation of dopamine. Dopamine hydrochloride was successfully oxidized to a quinone derivative by KMnO_4_, which then spontaneously polymerized into Dopa NPs. The morphological characteristics of the Dopa NPs were investigated through electron microscopy imaging. The synthesized Dopa NPs maintained a spherical shape and the size of the dried NPs was measured to be 83 ± 12 nm and 66 ± 11 nm, respectively (Figure S1A and Figure 2B) [20]. The chemical properties of the NPs were analyzed through FT-IR spectroscopy. The absorption band at 3254 cm^−1^ corresponds to the stretching vibrations of -OH and -NH groups in the Dopa NPs (Figure S1B). The broad peak was due to the presence of the catechol group in dopamine. The sharp peak at 1614 cm^−1^ was attributed to N-H bending vibrations. The peak at 1287 cm^−1^ indicates the C-O bond, while the peak at 1152 cm^−1^ corresponds to the stretching vibrations of C=C bonds within the ring structure [21]. The dispersion stability of the aqueous nanoparticle suspension was determined by the zeta potential. The zeta potential of Dopa NPs was −16 ± 1 mV, indicating the high stability of the NPs in the solution [22].

### 2.2. Cell Compatibility of Dopa NPs

The cell compatibility of Dopa NPs against Hep3B human hepatic cancer cells was investigated to assess their safety for tissue. The assay revealed that Dopa NPs were nontoxic to Hep3B up to a concentration of 100 µg/mL for 48 h of treatment (Figure S2). The treated cell maintained a viability over 100% when compared with the nontreatment control. These data indicate that Dopa NPs by themselves are non-toxic to Hep3B cells even at high concentrations and can be applied as a coating material for electrodes without any safety concerns.

### 2.3. Surface Analysis of Dopa NP-Coated Electrodes

**Figure 3:**
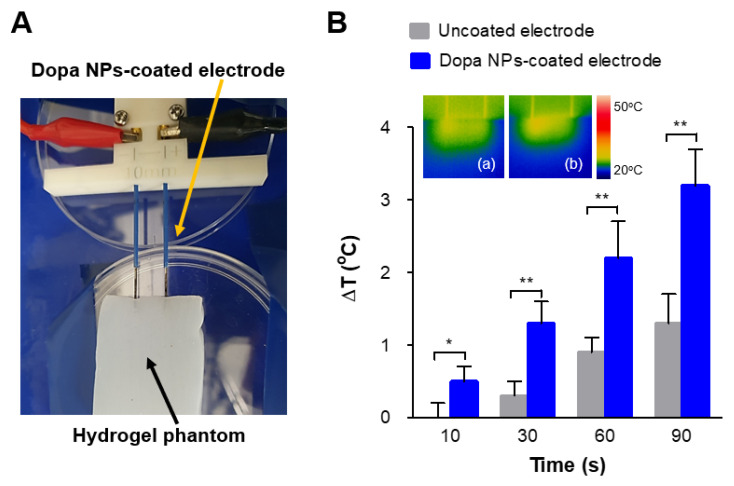
Hyperthermal effects of IRE induced by using Dopa NP-coated electrodes on a 2.4% konjac glucomannan hydrogel phantom model. (**A**) Experimental set-up for IRE (2 kV/cm, 100 µs-duration time, 90 pulses). (**B**) The extent of temperature induction under IRE with (**b**) Dopa NP-coated electrodes in comparison with (**a**) uncoated electrodes (unpaired-*t* test, * *p* < 0.05, ** *p* < 0.01, *n* = 3).

The stalk of the monopolar needle electrode was partially insulated using heat shrink tubing (shown in blue in Figure 3A). The non-insulated stalk and needle tip were coated with a mixture of Eudragit^®^ S100 and Dopa NPs using a dipping coating method (Figure 2A). Scanning electron microscope (SEM) images were utilized to observe the differences in the surface characteristics between the Dopa NP-coated electrode and the uncoated electrode. The uncoated electrode had a relatively smooth surface (Figure 2C), while the Dopa NP-coated electrode exhibited a rougher surface with a coating layer (Figure 2D). The SEM image analysis estimated the coating thickness of Dopa NPs on the electrode tip portion to be 10 ± 2.4 μm. The Dopa NPs were coated up to a length of 10 mm from the tip of the 21-G electrode. These electrodes were inserted 8 mm deep into the liver and pancreatic tissues of rabbits for the application of IRE pulses.

### 2.4. Enhanced Thermal Effect of IRE with Dopa NP-Coated Electrodes in a Phantom Model

We explored the potential of Dopa NP-coated electrodes to enhance thermal effects in response to IRE stimulation using a hydrogel phantom model (Figure 3A). As depicted in Figure 3B, the NP-coated electrode exhibited a significantly higher thermal effect compared to the uncoated electrodes. Specifically, the temperature increase observed during IRE with the NP-coated electrode was approximately 2.5 times higher than that achieved with the uncoated electrode.

As previously mentioned, the photo-induced thermal effect of Dopa NPs has been extensively studied across various models, including cancer tissues, infectious biofilms, and the skin layer of the Achilles tendon [23,24,25]. Interestingly, most reported data indicate that the thermal increase (ΔT) observed over a 90 s exposure period is typically less than 5 °C. Our findings demonstrate that Dopa NP-coated electrodes have the potential to increase temperatures under electric stimuli, which were conditionally adjusted for IRE therapy. This finding suggests that IRE therapy with NP-coated electrodes may improve the ablation effects on target cancer tissues.

Similar outcomes were noted when Dopa NPs were incorporated into a hydrogel phantom, as shown in Figure S2A. IRE (1 kV/cm, 0.6 ms duration time, 20 pulses) was applied to the alginate hydrogel phantom using an uncoated electrode (Figure S3A,B). The results showed that the temperature of the target area proportionally increased with escalating concentrations of Dopa NPs, ranging from 47 μg/mL to 188 μg/mL. The concentration-dependent thermal effects of Dopa NPs underscore their potential role in modulating the therapeutic impact of IRE, particularly in terms of hyperthermal ablation.

### 2.5. Enhanced In Vivo Thermal Effect of IRE with Dopa NP-Coated Electrodes

**Figure 4:**
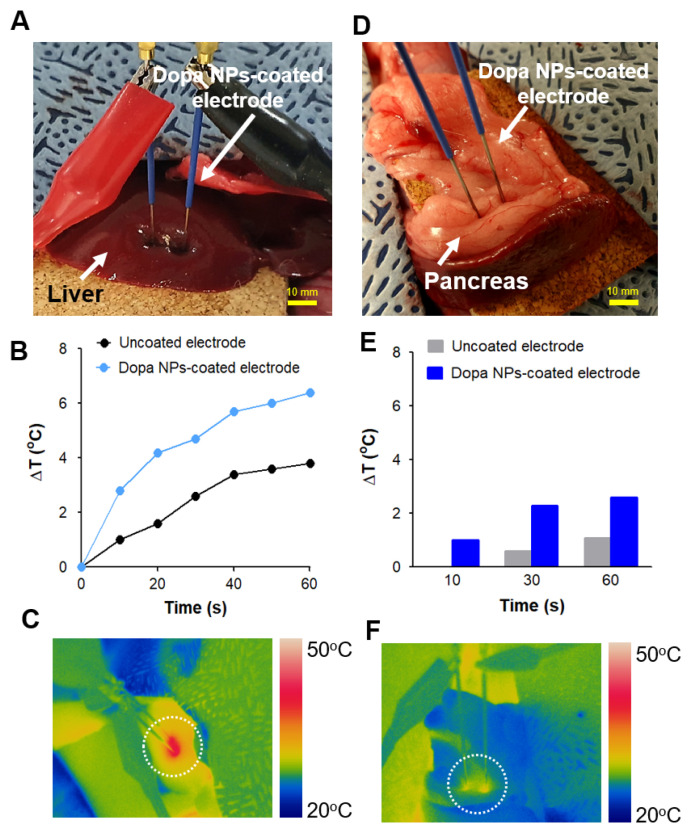
Thermal effect of Dopa NP-coated electrodes under IRE (1.5 kV/cm, 100 µs duration, 60 pulses) in an in vivo rabbit model. (**A**) IRE application to rabbit liver. (**B**) Thermal profiles in liver tissue during the IRE. (**C**) IR thermography image of IRE on hepatic tissue treated with Dopa NP-coated electrodes. (**D**) IRE application to rabbit pancreas. (**E**) Thermal profile of pancreatic tissue during IRE. (**F**) IR thermography image of IRE on rabbit pancreas treated with Dopa NP-coated electrodes. The white dotted circle indicates the thermal zone.

**Figure 5:**
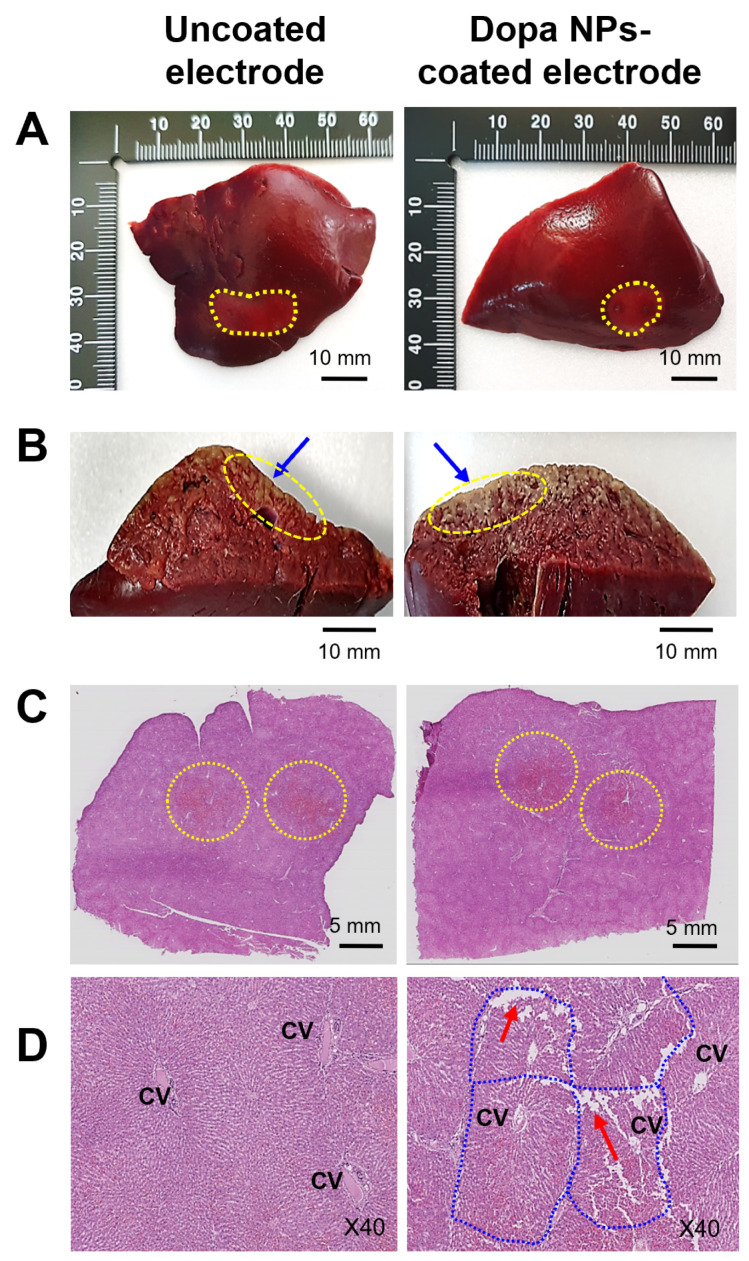
Histopathological observation of the ablated hepatic region after IRE (100 μs duration, 60 pulses) using an uncoated or Dopa NP-coated electrode. (**A**) Defined ablation zone (yellow dotted circles) after triphenyltetrazolium chloride (TTC) staining. (**B**) IRE ablation depth in TTC-stained tissue (blue arrows indicate ablated zone depth). (**C**) Whole-slide scan image of hematoxylin and eosin (H&E) stained tissue. (**D**) Partial disruption (red arrows) of hepatic lobular architecture in the tissue treated with the coated electrodes. The border of the hepatic lobule is marked with a blue dotted line. CV is an abbreviation for central vein.

Uncoated and Dopa NP-coated electrodes were individually introduced into the hepatic lobes of a rabbit model to monitor the enhanced temperature of target tissues during the IRE application (1.5 kV/cm, 100 μs duration time, 60 pulses) (Figure 4A). Although both the uncoated and NP-coated electrodes exhibited a similar surface ablation area of 161 mm^2^ (Figure 5A), the NP-coated electrode remarkably demonstrated a three-fold higher increase in temperature (ΔT) in the target area compared to the uncoated electrode (Figure 4B). The hepatic tissue treated with the NP-coated electrode exhibited a well-defined ablation zone, featuring a distinct pale circular–elliptical shape that was clearly discernible from the surrounding liver tissue (Figure 5A). In summary, the Dopa NP-coated electrodes demonstrated an additional thermogenic effect in vivo, elevating the temperature of the liver tissue by approximately 2–3 °C beyond the thermal response induced by IRE (Figure 4C).

We observed that the enhanced thermal effect of Dopa NP-coated electrodes is applicable to other organs (Figure 4D–F). The NP-coated electrode raised the tissue temperature in the rabbit’s pancreas by approximately 1–1.7 °C compared to the uncoated electrode under the same IRE conditions as those in hepatic tissue (Figure 4E,F).

### 2.6. Histological Observations

Histological analysis confirmed the heightened effectiveness of irreversible electroporation (IRE) when using Dopa NP-coated electrodes (Figure 5A–C). Whole-slide scans of the rabbit liver tissue post IRE (100 μs duration, 60 pulses) revealed distinct histopathological changes at the target site (Figure 5B). Notably, the hepatic lobe treated with NP-coated electrodes exhibited a greater disruption of the hepatic cord arrangement and lobular architecture compared to uncoated electrodes (Figure 5C).

In assessing the thermal ablation efficacy, we observed sinusoidal congestion around the central vein in the hepatic lobule in the IRE target area. Untreated tissue showed no congestion, while uncoated electrode-treated tissue displayed moderate congestion. Tissues treated with NP-coated electrodes exhibited severe sinusoidal congestion due to the infiltration and coagulation of erythrocytes in dilated hepatic sinusoids. The presence of a brown precipitate near the cathode insertion site of the NP-coated electrodes indicated localized cell damage from Dopa NP-induced heat generation (Figure S4). This concise analysis underscores the enhanced ablation effect of IRE with Dopa NP-coated electrodes.

## 3. Conclusions

This study reports the enhanced thermal ablation effect of IRE when combined with Dopa NPs. The Dopa NP-coated electrode demonstrated an elevated thermal effect on target tissues, attributed to enhanced electron transfer in IRE treatment; this resulted in significant red blood cell congestion and the partial disruption of the hepatic lobular structure in the hepatic region. Although our experiment was conducted on a normal tissue model, suggesting the need for more precise control experiments on tumor models to discern the thermal ablation effect on cancer tissue, we can conclude that the combined application of IRE and the conductive Dopa NPs suggests a promising avenue for clinical intervention. This approach may hold the potential to not only eradicate primary tumors but also prevent tumor metastasis, thereby reducing the risk of recurrence.

## 4. Materials and Methods

### 4.1. Materials

Dopamine hydrochloride (H8502, Sigma-Aldrich, St. Louis, MO, USA) was used as a basic material for nanoparticle synthesis. Potassium permanganate (KMnO_4_) was obtained from Yakuri Rure Chemicals Co. Ltd. (Kyoto, Japan). The WST-8 Cell Viability Assay Kit was supplied by Precaregene (Anyang, Republic of Korea). 2,3,5-triphenyltetrazolium chloride (T0520, TCI Co., Ltd., Tokyo, Japan) was used as an indicator dye to predict the ablation zone of the animal tissue. All reagents were of analytical grade and were used as received without further purification.

### 4.2. Synthesis of Polydopamine Nanoparticles

Dopa NPs were synthesized through the oxidation of dopamine hydrochloride with potassium permanganate (KMnO_4_, Yakuri Pure Chemicals, Co. Ltd., Tokyo, Japan). First, 120 mg of dopamine hydrochloride (H8502; Sigma-Aldrich Co., St. Louis, MO, USA) was dissolved in 54 mL of preheated distilled water. After it had dissolved, 6 mL of a 10 mM KMnO_4_ solution was gently added to the dopamine solution, followed by a 4 h reaction under vigorous stirring (200 rpm) at 50 °C. The product was collected and purified through centrifugation (5000 rpm for 10 min) and subsequently washed with distilled water several times. The purified Dopa NPs were dispersed in sterilized water, and the solid content was calculated.

### 4.3. Physicochemical and Morphological Analysis of Dopa NPs

The particle distribution and zeta potential of the synthesized Dopa NPs were determined at 25 °C by Dynamic Light Scattering (DLS) using a Zetasizer (Nano-ZS 90, Malvern Instruments, Worcestershire, UK). The functional groups of the synthesized nanoparticles were identified using a Fourier-transform infrared (FTIR) spectrometer (Spectrum Two^®^, Perkin-Elmer, MA, USA) that scanned the FTIR spectrum in the wavelength range of 400–4000 cm^−1^. The nanoparticle morphology was examined by a high-resolution scanning electron microscope (SEM, HR-SEM^®^ SU8010, Hitachi High-Tech Co., Tokyo, Japan) and a transmission electron microscope (TEM, JEM-2100F, JEOL Ltd., Tokyo, Japan) at a 15 kV accelerating voltage. Fifty nanoparticles were randomly selected from electron micrographs, and their sizes were determined using ImageJ software (version 1.52a: NIH, Bethesda, MD, USA).

### 4.4. Cytotoxicity Test of Dopa NPs against Hepatic Carcinoma Cell

The cytotoxicity of Dopa NPs on Hep3B cells (a human hepatoma cell line) and L929 cells (mouse fibroblast cell line) was assessed using a water-soluble tetrazolium (WST)-based assay. Cells (1 × 10^4^ cells/well) were seeded into a 96-well tissue culture plate containing Dulbecco’s Modified Eagle Medium (DMEM; Welgen, Seoul, Republic of Korea) supplemented with 10% FBS and 1% antibiotic-antimycotic. When cell growth reached approximately 80% confluency, the cells were treated with various NP concentrations (0, 5, 10, 25, and 50 µg/mL) in a humidified incubator (37 °C, 5% CO_2_) for 48 h. The cells were then treated with the CELLOMAXTM Viability Kit solution (Precaregene, Hanam, Republic of Korea), and their optical density was measured at 450 nm using a microplate reader (Infinite^®^ M200 PRO NanoQuant, TECAN. Ltd., Zurich, Switzerland). The cell viability was determined by calculating the percentage of relative absorbance values in the Dopa NP-treated cells compared to the untreated cells.

### 4.5. Preparation of Dopa NP-Coated Electrodes

We used a laboratory-fabricated needle electrode (21-gauge, 8 cm in length) equipped with a polyolefin insulating sheath and an acrylic electrode spacer. The needle electrodes were fabricated in the laboratory using acupuncture needles with closed conical stainless steel active tips. Eudragit^®^ S100 (Evonik Röhm GmbH, Darmstadt, Germany) was employed as a coating additive to enhance the adhesion of nanoparticles to the surface of the needle electrode. Eudragit^®^ S100 was dissolved in a solvent (acetone: ethanol = 1:1, *v*/*v*) at a concentration of 0.2% (*w*/*v*). The electrodes were dipped in a 0.1% (*w*/*v*) Dopa NP-resuspended Eudragit solution and air-dried. This coating process was repeated for 10 or more cycles. The coated electrodes were examined for their surface characteristics using a Mini-SEM (SNE-3200M, SEC Co., Ltd., Suwon, Republic of Korea) at an acceleration voltage of 10 kV, an emission current of 110 µA, and a magnification of × 350.

### 4.6. Monitoring of Hyperthermal Effects of IRE on Hydrogel Phantom Model

The experiments were conducted using Dopa NP-coated electrodes and standard probes to compare the ablation and hyperthermal effects across various models. First, we investigated the ability of Dopa NP-coated electrodes to induce hyperthermal effects under electrical stimulation using a 2.4% konjac glucomannan hydrogel phantom (Samjin Food Co., Ltd., Chungbuk, Republic of Korea). The distal end of the electrodes was inserted into each phantom. The proximal portion of the electrode was connected to a direct current pulse generator (ECM 830 square wave electroporation system, BTX Genetronics, CA, USA), which delivered monophasic pulses to the phantom matrix. We compared the thermal profiles between the two electrodes during electrical stimulation (2 kV/cm, 100 µs duration time, 90 pulses, 2 mm active tip length) using an Optris^®^ Xi 400 infrared camera (Optris GmbH, Berlin, Germany).

### 4.7. Animal Care and Ethical Approval

The animal care and experimental procedures were conducted in accordance with the protocols approved by the Institutional Animal Care and Use Committee (Approval No. INHA 200820-713). Normal New White Zealand rabbits (3 males, 2.8 ± 0.5 kg in weight) were obtained from DBL Co., Ltd. (Chungbuk, Republic of Korea). They were housed in individually ventilated cages with free access to water and food at the laboratory animal facility of Inha Bio-Medical Research Institute (Incheon, Republic of Korea). After an acclimation period of one week or more in temperature-controlled rooms, the animals were used for the IRE experiments.

### 4.8. In Vivo Animal Study

The animal study was performed using a normal healthy rabbit model. General anesthesia was induced with an intramuscular injection of alfaxalone (Alfaxan^®^ Multidose, Jurox Animal Health, Rutherford, Australia, 2 mg/kg) prior to IRE application. Intraoperative anesthesia was maintained by administering 1.5–2% isoflurane (Ifran^®^, Hana pharm. Co. Ltd., Seoul, Republic of Korea) through inhalation using a veterinary anesthesia system (SV-3000 anesthesia workstations, Soar Medical Tech Co. Ltd., Taipei City, Taiwan). A midline laparotomy was performed on the anesthetized rabbits to expose either their hepatic lobe or pancreas. Uncoated and Dopa NP-coated needle electrodes (21-gauge) were inserted into each target organ, and IRE (1.5 kV/cm, 100 µs duration time, 60 pulses) was applied using a pulse electroporator (ECM 830). The temperature changes in hepatic or pancreatic tissues during the delivery of IRE pulses were recorded using an Optris^®^ Xi 400 infrared camera (Optris GmbH, Berlin, Germany). Additionally, we visually confirmed the occurrence of muscle contractions during IRE application and assessed the survival status of the animals immediately after IRE.

### 4.9. Histopathological Examination of IRE-Treated Liver in Rabbit Model

The rabbits were sacrificed at least 2.5 h post-IRE procedure to examine the histopathological characteristics of the hepatic lobes with three different treatments (untreated, IRE with uncoated electrodes, and IRE with Dopa NP-coated electrodes). The harvested hepatic lobes were stained with 2% TTC (2,3,5-triphenyltetrazolium chloride) solution to observe the ablated zone. The tissue specimens were fixed in 10% neutral buffered formalin (StatLab, McKinney, TX, USA) and stored at 4 °C until further tissue processing. After cutting the tissue perpendicular to the electrode insertion direction, the tissue sections (5 µm in thickness) were stained with hematoxylin and eosin (H&E) following conventional protocols. Whole-slide images of the tissue were obtained using a digital whole-slide scanner (Leica SCN 400, Leica Biosystems Inc., Wetzlar, Germany). We examined the hepatic lobule architecture, sinusoidal dilation, sinusoidal congestion, size of hepatocyte nuclei, and other notable histological changes in the vicinity of the anode and cathode insertion sites. High-resolution microscopic images (magnification: ×40, ×100, and ×400) were acquired using the iSolution Lite imaging analysis software program (ver. 9.1, IMT i-Solution Inc., Vancouver, BC, Canada).

### 4.10. Statistical Analysis

The data obtained from experiments with three or more replicates were represented as mean ± SD. Statistical analysis was conducted using Microsoft Excel and GraphPad Prism software (ver. 7.0, GraphPad Software Inc., La Jolla, CA, USA), and a *p*-value < 0.05 was considered statistically significant. The statistical significance of the hydrogel phantom study (*n* = 3) and cytotoxicity test (*n* = 4) was analyzed using one-way ANOVA for comparisons involving three or more groups, while unpaired t-tests were employed for comparisons between two groups.

## Data Availability

The data that support the findings of this study are available from the corresponding author upon reasonable request.

## Supplementary Materials

The following supporting information can be downloaded at: https://www.mdpi.com/article/10.3390/ijms25084317/s1.

## References

[1] Gajewska-Naryniecka A., Szwedowicz U., Łapińska Z., Rudno-Rudzińska J., Kielan W., Kulbacka J. (2023). Irreversible Electroporation in Pancreatic Cancer—An Evolving Experimental and Clinical Method. Int. J. Mol. Sci..

[2] Davalos R.V., Mir L., Rubinsky B. (2005). Tissue ablation with irreversible electroporation. Ann. Biomed. Eng..

[3] Wah T.M., Lenton J., Smith J., Bassett P., Jagdev S., Ralph C., Vasudev N., Bhattarai S., Kimuli M., Cartledge J. (2021). Irreversible electroporation (IRE) in renal cell carcinoma (RCC): A mid-term clinical experience. Eur. Radiol..

[4] Woeste M.R., Shrestha R., Geller A.E., Li S., Montoya-Durango D., Ding C., Hu X., Li H., Puckett A., Mitchell R.A. (2023). Irreversible electroporation augments β-glucan induced trained innate immunity for the treatment of pancreatic ductal adenocarcinoma. J. Immunother. Cancer.

[5] Guo Y., Zhang Y., Klein R., Nijm G.M., Sahakian A.V., Omary R.A., Yang G.-Y., Larson A.C. (2010). Irreversible electroporation therapy in the liver: Longitudinal efficacy studies in a rat model of hepatocellular carcinoma. Cancer Res..

[6] Jourabchi N., Beroukhim K., Tafti B.A., Kee S.T., Lee E.W. (2014). Irreversible electroporation (NanoKnife) in cancer treatment. Gastrointest. Interv..

[7] Zhao J., Qiao Y., Zhou M., Wallace M., Gupta S., Li C., Melancon M.P. (2015). Antitumor efficacy of irreversible electroporation and doxorubicin-loaded polymeric micelles. ACS Macro Lett..

[8] Ahmed M., Brace C.L., Lee F.T., Goldberg S.N. (2011). Principles of and advances in percutaneous ablation. Radiology.

[9] Jiang Y., Jenjob R., Yang S.-G. (2022). Enhanced Therapeutic Potential of Irreversible Electroporation under Combination with Gold-Doped Mesoporous Silica Nanoparticles against EMT-6 Breast Cancer Cells. Biosensors.

[10] Kim D., An J., Surendran S., Lim J., Jeong H.-Y., Im S., Kim J.Y., Nam K.T., Sim U. (2023). Synergistic effect of Polydopamine incorporated Copper electrocatalyst by dopamine oxidation for efficient hydrogen production. J. Colloid Interface Sci..

[11] Park J., Moon H., Hong S. (2019). Recent advances in melanin-like nanomaterials in biomedical applications: A mini review. Biomater. Res..

[12] Kang S., Baskaran R., Ozlu B., Davaa E., Kim J.J., Shim B.S., Yang S.G. (2020). T_1_-Positive Mn^2+^-Doped Multi-Stimuli Responsive poly(L-DOPA) Nanoparticles for Photothermal and Photodynamic Combination Cancer Therapy. Biomedicines.

[13] Chao B., Jiao J., Yang L., Wang Y., Jiang W., Yu T., Wang L., Liu H., Zhang H., Wang Z. (2023). Application of advanced biomaterials in photothermal therapy for malignant bone tumors. Biomater. Res..

[14] Liu Y., Ai K., Lu L. (2014). Polydopamine and its derivative materials: Synthesis and promising applications in energy, environmental, and biomedical fields. Chem. Rev..

[15] Siciliano G., Monteduro A.G., Turco A., Primiceri E., Rizzato S., Depalo N., Curri M.L., Maruccio G. (2022). Polydopamine-coated magnetic iron oxide nanoparticles: From design to applications. Nanomaterials.

[16] Liu L., Ma S., Pei Y., Xiong X., Sivakumar P., Singler T.J. (2016). Regulation of the Deposition Morphology of Inkjet-Printed Crystalline Materials via Polydopamine Functional Coatings for Highly Uniform and Electrically Conductive Patterns. ACS Appl. Mater. Interfaces.

[17] Chalmers E., Lee H., Zhu C., Liu X. (2020). Increasing the Conductivity and Adhesion of Polypyrrole Hydrogels with Electropolymerized Polydopamine. Chem. Mater..

[18] Lu J., Cai L., Dai Y., Liu Y., Zuo F., Ni C., Shi M., Li J. (2021). Polydopamine-Based Nanoparticles for Photothermal Therapy/Chemotherapy and their Synergistic Therapy with Autophagy Inhibitor to Promote Antitumor Treatment. Chem. Rec..

[19] Zhang Z., Zhang J., Tian J., Li H. (2021). A polydopamine nanomedicine used in photothermal therapy for liver cancer knocks down the anti-cancer target NEDD8-E3 ligase ROC1 (RBX1). J. Nanobiotechnol..

[20] Ho C.-C., Ding S.-J. (2013). The pH-controlled nanoparticles size of polydopamine for anti-cancer drug delivery. J. Mater. Sci. Mater. Med..

[21] Luo H., Gu C., Zheng W., Dai F., Wang X., Zheng Z. (2015). Facile synthesis of novel size-controlled antibacterial hybrid spheres using silver nanoparticles loaded with poly-dopamine spheres. Rsc Adv..

[22] Tang Z., Ma Z. (2017). Ultrasensitive amperometric immunoassay for carcinoembryonic antigens by using a glassy carbon electrode coated with a polydopamine-pb (ii) redox system and a chitosan-gold nanocomposite. Microchim. Acta.

[23] Zhou Z., Li S., Gong X. (2023). Polydopamine Nanoparticles-Based Photothermal Effect Against Adhesion Formation in a Rat Model of Achilles Tendon Laceration Repair. Int. J. Nanomed..

[24] Zhu Z., Su M. (2017). Polydopamine Nanoparticles for Combined Chemo- and Photothermal Cancer Therapy. Nanomaterials.

[25] Gao R., van der Mei H.C., Ren Y., Chen H., Chen G., Busscher H.J., Peterson B.W. (2021). Thermo-resistance of ESKAPE-panel pathogens, eradication and growth prevention of an infectious biofilm by photothermal, polydopamine-nanoparticles in vitro. Nanomedicine.

