# Supplementary material for: The Induction of Combined Hyperthermal Ablation Effect of Irreversible Electroporation with Polydopamine Nanoparticle-Coated Electrodes
Source: Int J Mol Sci. 2024 Apr 13;25(8):4317. doi: 10.3390/ijms25084317 (PMC11050635; doi:10.3390/ijms25084317)
Supplement: Supplementary file 1 [file ijms-25-04317-s001.zip › ijms-2902554-supplementary.pdf]

Supplement data

The enhanced thermal ablation effect of irreversible electroporation with dopa nanoparticle-coated electrodes

Sung-Min Jeon [1,2], Enkhzaya Davaa [1], Ratchapol Jenjob [1], Chiravoot Pechyen [3,4], Sitakan Natphopsuk [3,5], Seok Jeong [6], Hye Jin Yoo [1,2,*] and Su-Geun Yang [1,2,*]

[1] Department of Biomedical Science, BK21 FOUR Program in Biomedical Science and Engineering, Inha University College of Medicine, Incheon 22212, Republic of Korea
[2] Inha Institute of Aerospace Medicine, Inha University College of Medicine, Incheon 22332, Republic of Korea
[3] Thammasat University Center of Excellence in Modern Technology and Advanced Manufacturing for Medical innovation, Thammasat University, Pathumthani 12120, Thailand
[4] Department of Materials and Textile Technology, Faculty of Science and Technology, Thammasat University, Pathumthani 12120, Thailand
[5] Chulabhorn International College of Medicine, Thammasat University, Pathumthani 12120, Thailand
[6] Division of Gastroenterology, Inha University Hospital, Inha University College of Medicine, Incheon 22332, Republic of Korea
* Correspondence: hyejin_yoo@inha.ac.kr (H.J.Y.); sugeun.yang@inha.ac.kr (S.-G.Y.)

Keywords: Irreversible electroporation (IRE), Polydopamine nanoparticles, Hyperthermal effect, Cancer ablation.

Monitoring of hyperthermal effects of IRE on hydrogel phantom model

We studied the temperature changes of alginate hydrogel phantoms induced by varying concentrations of NPs under electrical stimulation. The NPs-mixed cylindrical alginate hydrogel phantoms were prepared using the extrusion gelation method. We prepared 4% (w/v) alginate solutions with different concentrations of NPs (0, 47, 188, and 750 μg/mL) by adding sodium alginate (250 cps, 25°C, Sigma-Aldrich, USA) and NPs to distilled water at 50°C. The mixed hydrogel (6 mL) was transferred to a disposable syringe assembled with a 23-gauge needle and allowed to stand at 50°C until all residual bubbles were removed. A cylindrical phantom was formed by dropping the mixture into a 2.5% (w/v) $CaCl_2$ cross-linking solution (150 mL) at a rate of 0.15 mL/sec. The curing process in the cross-linking solution and the subsequent gentle rinsing process to remove excess residual calcium ions were performed twice. A pair of 23-gauge needle electrodes was inserted into the central region of the alginate phantom specimen (70 mm long).

Monophasic pulses (1kV/cm electric field strength, 0.6 ms duration time, 20 pulses) were delivered to it using a pulse electroporator (ECM 830 Square Wave Electroporation system, BTX Harvard Apparatus, Holliston, MA, USA). The real-time temperature changes of the phantom were monitored using an infrared camera (Optris® Xi 400, Optris GmbH, Berlin, Germany).

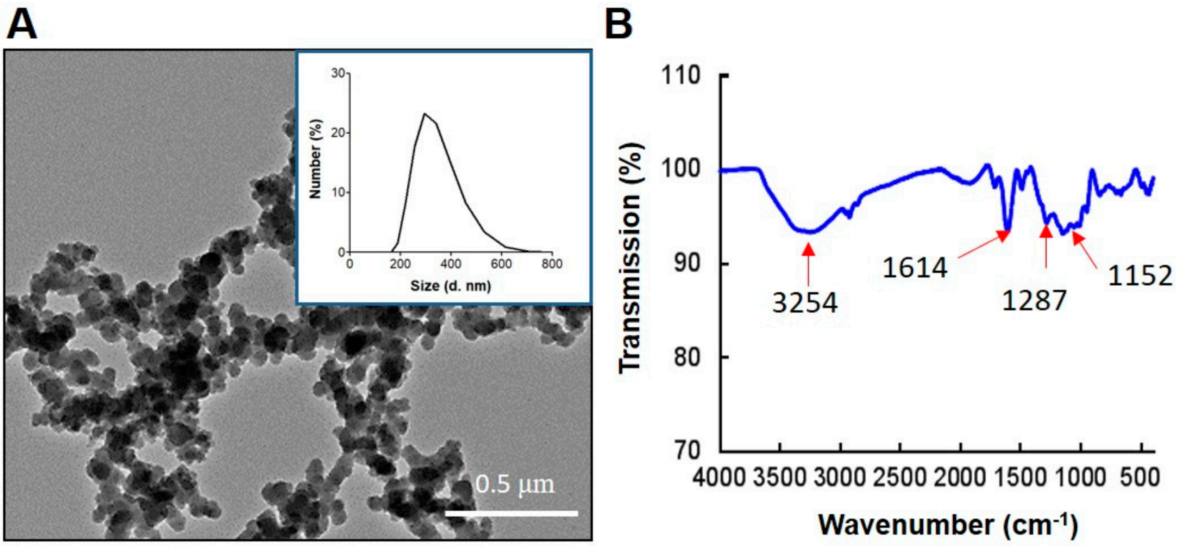

Figure S1. (A) TEM image of Dopa NPs (inset: hydrated particle size distribution of Dopa NPs), and (B) FT-IR spectrum of Dopa NPs.

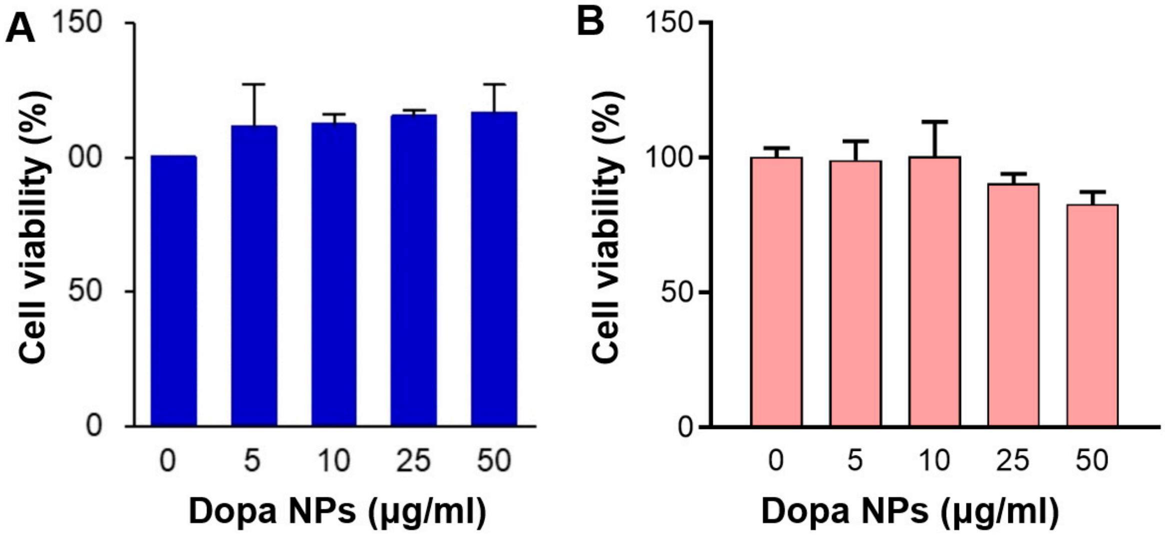

Figure S2. *In vitro* cytotoxicity of Dopa NPs against (A) Hep3B cells and (B) L929 cells. The cell viability was determined after treatment with different NPs concentrations for 48 h using the water-soluble tetrazolium assay.

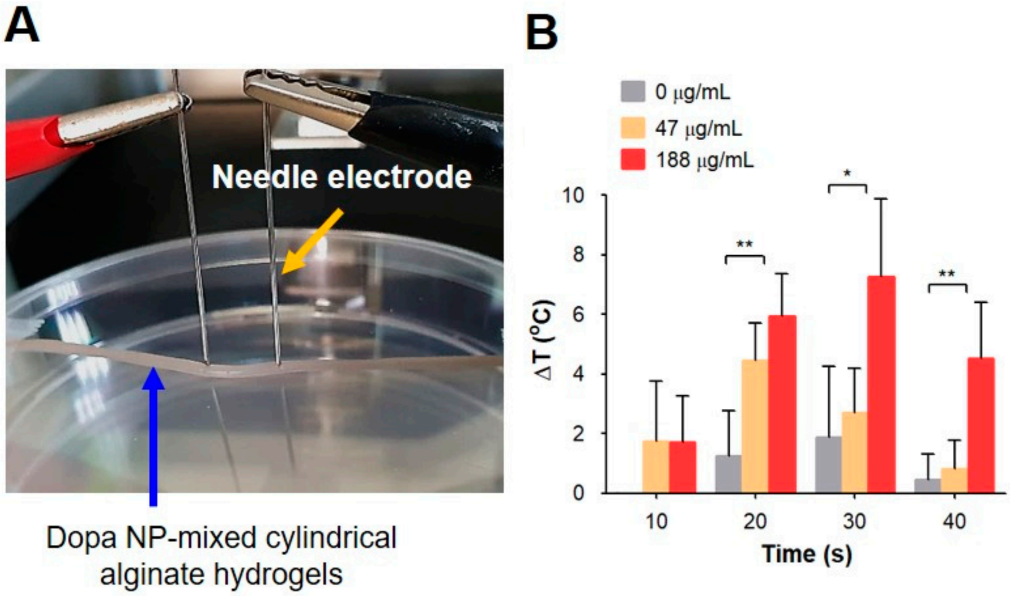

Figure S3. Thermal effect of Dopa NPs according to Dopa NPs content in cylindrical alginate hydrogel during irreversible electroporation. (A) Experimental setup for monitoring the surface temperature of NPs-mixed alginate hydrogel phantom. (B) Thermal profile of the NPs-mixed phantom with electroporation (1kV/cm, 0.6 ms-duration time, 20 pulses) over time. Statistically significant value compared with control group data by ANOVA test (*P<0.05,**P<0.01, n=3).

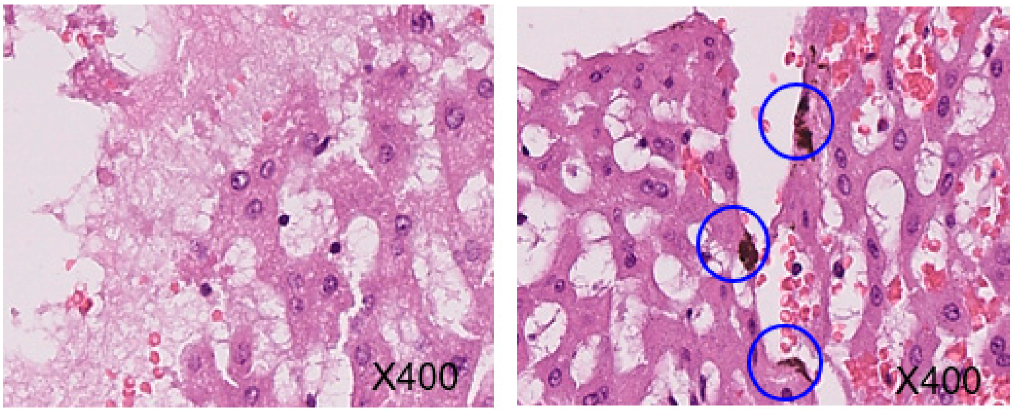

Figure S4. Histopathological observation of the ablated hepatic region after IRE treatment (100 μs-duration time, 60 pulses) using an uncoated or Dopa NPs-coated electrodes. Brownish deposits (blue circles) observed in the tissue treated with the coated electrodes.
